# Supplementary material for: Automatic methods of hoof-on and -off detection in horses using wearable inertial sensors during walk and trot on asphalt, sand and grass
Source: PLoS One. 2021 Jul 26;16(7):e0254813. doi: 10.1371/journal.pone.0254813 (PMC8312981; doi:10.1371/journal.pone.0254813)
Supplement: Attachment — Submitted filename: Response to Reviewers.docx [file pone.0254813.s001.docx]

| **Journal Requirements** | | |
| --- | --- | --- |
| **1** | Review your reference list to ensure that it is complete and correct. | Reference list has been reviewed |
| **2** | Ensure that your manuscript meets PLOS ONE's style requirements, including those for file naming | The submission has been reviewed |
| **3** | Provide an amended Competing Interests Statement that explicitly states this commercial funder, along with any other relevant declarations relating to employment, consultancy, patents, products in development, marketed products, etc. Within this Competing Interests Statement, please confirm that this does not alter your adherence to all PLOS ONE policies on sharing data and materials by including the following statement: "This does not alter our adherence to PLOS ONE policies on sharing data and materials.” | The Competing Interests Statement has been updated to read:  *“The research of EVB is funded by Worldbase Ltd. and this study was also supported by the UK EPSRC (EP/K03877X/1, EP/S032940/1, https://epsrc.ukri.org). The funders had no role in study design, data collection and analysis, decision to publish, or preparation of the manuscript. The commercial funder Worldbase Ltd. is a manufacturing company, specialising in agricultural machinery and currently has no products or services, existing or in design, related to the content of this research. This does not alter our adherence to PLOS ONE policies on sharing data and materials.”*  This response has been added to the Cover Letter, as per the editors instruction. |
| **Reviewer 1- Comments** | | |
| **1** | Lack of accurate definition of what type of event they’re looking at | Hoof-on and off events are defined as the instants when the hoof comes into contact with the ground, at the onset of the stance duration, and when the hoof is lifted from the ground, following break-over, respectively. These definitions are as per Witte et al. (2004) (1).  Following your comments, we have added a definition of how we use the term gait event and what we consider to be a hoof-on and hoof-off, including a schematic (Fig 2):  “*The gait events (Fig 2) were defined as the instants in the stride cycles when the hoof first comes into contact with the ground at the onset of the stance phase (hoof-on) and when it is lifted from the ground, following break-over (hoof-off)* (1)*”* |
| **2** | Found the title misleading- I expected algorithm which differentiated between walk and trot, while it only detects when foot is on or off within a gait | We hope, if we have correctly understood the reviewer’s comment, that confusion has been avoided by modifying the title as:  *“Automatic methods of hoof-on and -off detection in horses using wearable inertial sensors during walk and trot on asphalt, sand and grass”* |
| **3** | Sometimes use values as % and sometimes in ms to compare methods without explaining why | Our rationale here was that, when reporting for an individual event, we would express the error in ms; when reporting the errors incurred in calculating a proportion of a gait cycle (stance duration) we chose to express the error as a percentage. We have added explicit detail regarding this to the methods section:  “*For individual gait events, the errors were expressed in ms*”  And, when describing presentation for the errors incurred in calculating stance duration:  “*Errors were expressed as percentages of the total stride duration*” |
| **4** | Some statistical results summary confusing as not using the common standards | We have updated the relevant table to reflect the reviewer’s comments, now using ** and *** to indicate p<0.01 and p<0.001, respectively. |
| **Reviewer 1- Specific Comments** | | |
| **1** | title: you say gait event detection but you don't really detect the gait (like is it walk or trot), you detect whether the off is on or off. I would adapt the title accordingly | Following the reviewer’s comments, we have altered the title and hope that the meaning is clearer:  *“Automatic methods of hoof-on and -off detection in horses using wearable inertial sensors during walk and trot on asphalt, sand and grass”* |
| **2** | 56: Keegan always uses a gyroscope on the pastern in his papers | Thank you for your comment. We have studied much of Dr. Keegan’s work and indeed he does but his methods for detecting hoof-on and -off from these sensors are not publicly available (presumably due to commercial sensitivities with Dr Keegan heading up the team who developed the Lameness Locator, to which we refer). To reflect your comment, we have amended the text to:  *“Several previous studies have used inertial devices attached at the level of the pasterns to identify strides* (2) (3) (4)*, and there is a commercial system which uses these methods (Lameness Locator™, Equinosis, Columbia). However, to the authors’ best knowledge, there are no published methods for specific gait event detection which are publicly available.”* |
| **3** | 46-58: I would be more accurate in what do you include in "gait event" please. | Several additions have been made to the manuscript, to better define our use of the term ‘gait events’. These appear in the first paragraph of the introduction:  “*These gait events are the instants in a gait cycle when the hoof first comes into contact with the ground (hoof-on) and that when it is first lifted fully from the ground (hoof-off)* (1)*”*  And the first paragraph of section 2.3:  “*The gait events (Fig 2) are defined as the instants in the stride cycles when the hoof first comes into contact with the ground at the onset of the stance phase (hoof-on) and when it is lifted from the ground, following break-over (hoof-off)* (1)  After which an illustration of hoof-on and -off can be found (Fig 2). |
| **4** | 68: specify more precisely what events are you interested in | We hope that our more explicit definition of ‘gait events’, earlier in the section, mean that this is now clear.  “*These gait events are the instants in a gait cycle when the hoof first comes into contact with the ground (hoof-on) and that when it is first lifted fully from the ground (hoof-off)* (1) |
| **5** | 73: you have to specify somewhere that you are going to provide mean (sd) values | Thank you. We have now amended the referenced lines to:  *“Eleven horses (eight geldings and three mares, of mean(sd) heights and ages of 154(21)cm and 12(8)years)”*  And later added another definition of the structure the first time it is used in the presentation of results:  *“…the two methods were equally successful with M1_p_ detecting events slightly late by a mean(sd) delay of 2(10)ms, and M2_p_ slightly early, -1(10)ms…”*  In table 1 we had already defined *Mean(sd)* and have left this as was. |
| **6** | 86: are we sure that the pastern attachment doesn't disturb the horse's motions? | The Shimmer sensors which we used were small enough to not impede the action of the fetlock joint. Hence, we would not expect the methods to cause any more annoyance than the usual wearing of pastern wraps, without sensor attachment. The horses we employed were all well used to wearing pastern wraps, brushing boots and similar leg protection and we did not observe any signs of discomfort. |
| **7** | 99: what do you mean by "timing of gait events"? Hence we need a proper definition of what do you consider as a gait event, is it the stride duration? the moment where the hoof is on or off? | The ‘timing of gait events’ is the instant when each gait event occurs. We hope that this has now been sufficiently clarified by our better definition of what we mean by gait events:  “*The gait events (Fig 2) are defined as the instants in the stride cycles when the hoof first comes into contact with the ground at the onset of the stance phase (hoof-on) and when it is lifted from the ground, following break-over (hoof-off)* (1)  And Fig 2: |
| **8** | 106-108: I am not sure to understand why you provide the accuracy of the method in "ms" while you are doing some gait detection so I would have expected a percentage of error. Can you explain me a bit better what does this error in "ms" represent? | When referring to the detection of single events (hoof-on and -off) we express errors in terms of ms because we are detecting specific instants in time (the occurrence of the events). Later, when looking at the error incurred by the methods when used to calculate stance duration (a proportion of the stride cycle) we express the errors in percentage of a total stride duration (%). |
| **9** | 107: is the reference method only use "ms" then you should use this unit consistently across your whole paper | Our paper does not concern comparing the errors incurred by our methods to those incurred by the reference method; rather, the performance of our methods compared to others we employed from the literature (5) (6) and the performance of each of our novel methods compared to each other. The errors reported for the reference method (7) are provided using their units so that the reader is aware of how accurate this method was. |
| **10** | 190: LoA have not been stated here prior to its use later in the paper | Thank you for this observation. When the concept is first introduced, we have now clarified with the phrase:  “…*was quantified by the limits of agreement (LoA), with upper and lower limits of agreement (ULoA and LLoA) calculated as per …”* |
| **11** | 287: Be careful you didn't perform an equivalence test, the absence of significance in a difference test don't allow you to refer to them as equivalent. You can say that they have the same performance but not that they are equivalent. | Thank you for this comment, we have altered the wording accordingly and hope the meaning is now unambiguous:  “…*suggesting methods had the same performance for all individuals.*” |
| **12** | 308: it might worth defining what is a stride cycle. | A definition of stride cycle has now been added to the beginning of section 2.3, following the explicit definition of the gait events:  “*A stride cycle refers to one full cycle of gait, from one hoof-on to the subsequent hoof-on*.” |
| **13** | l.324: for all methods you report an accuracy in percentage, why for M4c you report as ms? it is not directly comparable to percentages | The reason for this was that the paper from which M4_c_ is taken (5) errors are reported in ms. Indeed, the values in the line to which you refer (“*-5.4ms to 14.2ms*”) were taken directly from the paper. Hence, when comparing our methods to this method, and the accuracy achieved when we used this method compared to what the authors had reported (5), we used the error in ms.  Furthermore, as reported previously, we reported errors for individual events in terms of ms and errors for stance duration calculation in %.  When comparing the errors we obtained applying M3_c_ to our data to those reported in the paper from which it was taken (6), we reported the errors as percentage of a stride duration, to allow easy comparison to the original paper, as this was the unit used there. Upon your suggestion, we have now been more explicit about this in the discussion section, in the hope of better guiding the reader:  “*The errors incurred by M3_c_ and M4_c_ in this study were substantially larger than those reported in the literature. In the source paper, M3_c_ was reported to incur a bias of 0.6% and 0.1% of a stride cycle for forelimb hoof-on and -off events at trot* (6)*. In the present application, for hoof-on and -off, the method incurred errors of -26ms and 62ms, respectively, equivalent to -3% and 7% of a stride cycle. These errors are some 5 and 70 times greater than in the original source*.” |
| **14** | l.355-357: again you are mixing accuracy in percentages with accuracy in ms. Can't you decide on only one way of reporting the accuracy across the whole paper? | Thank you for pointing this out. We have now altered the errors to ms to be in keeping with our rationale for reporting errors for individual events in terms of ms and for calculating stance duration in terms of %:  “…*slightly worse for most events even if still having overall good accuracy (-7 to 15ms) and precision (9 to 38ms)*” |
| **15** | l.368-370: Be careful you didn't perform an equivalence test, the absence of significance in a difference test don't allow you to refer to them as equivalent. You can say that they have the same performance but not that they are equivalent. | We have altered the text in the hope there is now no ambiguity:  “….*methods had an equivalent performance on all surfaces…*” |
| **16** | limitation: M4c: if we have to measure for each sensor and each horse the angle to be able to do the calculation isn't this method highly time consuming and won't be able to be automated? | Thank you for this very engaging question. The implementation of this method was fully automated and the means of achieving that are comprehensively detailed in the paper and supporting material of Bragança et al. (5). We also consulted with the lead author several times to discuss (hence the acknowledgements). The angles, which are calculated directly from data signals, are in fact only used in an initial step to estimate where the individual stride cycles are in the data before a more detailed search is carried out to locate the instants of hoof-on and -off. |
| **17** | Table 3: I would rather use the commonly used pvalue scale which * = <0.05, **=<0.01 and ***= <0.001 in order to facilitate the readability and prevent going back to the legend all the time | Thank you for this recommendation, we have now used your suggested notation and hope that the meaning is now clearer. |
| **18** | Figure p34: on Bland and Altman graphs, you can go further in the interpretation of the results than just looking at the average error value and its confidence interval. For example, we can see for hindlimb at trot: below 300ms the method has a tendency to overestimate values for some strides on grass and sand while it underestimates them after 300ms. While at walk there is fewer underestimations. | Thank you for this observation, we have now included this analytical comment to the manuscript:  “*In the case of the hindlimbs at trot, the Bland-Altman plots appear to show that the method tended to underestimate the stance duration for mean stance durations of over 300ms. However, the points above 300ms which fell below the LLoA (10 points) are very few compared to the points which fell within the LoA. Conversely, the Bland-Altman plot for the hindlimbs at trot suggest that the method tended more frequently to overestimate the stance duration for mean stance durations of below 300ms. However, the points here falling above the ULoA, compared to those falling within the LoA, are again only a small minority. These two observations could warrant further testing to determine whether there is a trend between the duration of the stance phase and whether the methods tend to more frequently under- or overestimate the stance duration*.” |
| **Reviewer 2- Comments** | | |
| **1** | I am curious as to why the authors relied on event detection rather than using machine learning or neural networks, as machine learning is becoming more common in this field. The accuracy reported here is acceptable, but it would be interesting to see if increased accuracy could be gained via different techniques. A brief note somewhere on the decision to use the selected algorithms rather than a machine learning approach would be useful. | We do not think our cohort of 11 horses was large enough for machine learning techniques to be effectively employed. Indeed, in a recent publication into gait classification using neural networks, data from 120 horses was used for model training and testing (8). Nevertheless, following your interesting comment, it is something we could explore in future work when more data will be available, thank you. |
| **2** | The introduction would benefit from more detail on the potential usefulness of gait event detection (e.g., equitation related research, lameness diagnosis etc.) for those not familiar with the field.  Overall I would like to see more in depth assessment of the existing literature in both the introduction and the discussion. | We thank you for raising this point. We have added an additional paragraph to the introduction to suggest a number of uses for gait event detection, as per your suggestion:  “*Methods to accurately detect gait events are valuable in various applications including performance analysis and lameness quantification. For instance, an increase in positive diagonal advanced placement (in which the hoof-on of the hindlimb precedes that of the contralateral forelimb) has been found to be an indicator of superior gait quality in advanced dressage horses* (9) *and approved Warmblood stallions* (10)*. Furthermore, the timing of gait events can be used to calculate the suspension of flying gaits, in which all four hooves are off the ground simultaneously. Studies into lameness detection have consistently found there to be a link between a statistically significant reduction in suspension phase and the presence of lameness* (11) (12) (13)*.”*  Throughout, we have added additional references and appraisal of the literature. In particular, in the discussion we have added two additional paragraphs to give the errors some biological context:  “*It has previously been reported that mild and moderate forelimb lameness can cause reductions in trot stride duration of 11ms and 31ms, respectively* (13)*. The mean errors incurred by the described pastern-based methods to detect hoof-on events at trot (-2ms for forelimbs and 1ms for hindlimbs) are small enough that the methods would be reliable for measuring such changes*”  “*In the literature, it was reported that unilateral forelimb lameness increased the stance phase of the ipsilateral hindlimb by 1.3% and that of both forelimbs by 2.3% in horses trotting on a treadmill* (12)*. The mean error in trotting stance durations calculated using the described methods range from 0.1 to 1.16% of a stride duration, suggesting that the methods are sufficiently accurate to detect these lameness-dependent changes”* |
| **3** | Regarding the use of force plates, I agree that they are impractical under most circumstances– but there have been limited studies using force plates in the field (e.g., Self Davies et al. 2019) but the complexity of embedding force plates under tracks is an obvious disadvantage. | Thank you for this point. To present a more balanced appraisal of force plate use, we have amended part of the introduction to read:  “*However, force plates not only come with extremely high costs but limit the number of strides which can be analysed, requiring a single foot to be placed fully on the plate for reliable analysis. Instrumented treadmills* (14) *have been used to overcome the latter point but these are often unsuitable for simulating real-world scenarios. Although a very limited number of studies have now used force plates in field conditions* (15) (16) *the obvious complexities of integrating them into surfaces limit wider adoption of such methods. These factors make force plates unsuitable for collecting data under field conditions.”* |
| **4** | In the discussion and/or limitations, some attention should be paid to how acceptable the reported errors are from a biological standpoint. The errors are small, but depending on the intended use of this technology, the errors may limit the usefulness of the sensors. | Thank you for this valuable comment. To offer some context of how significant the errors are in terms of biological application, we have added the following lines to the discussion:  *“It has previously been reported that mild and moderate forelimb lameness can cause reductions in trot stride duration of 11ms and 31ms, respectively* (13)*. The mean errors incurred by the described pastern-based methods to detect hoof-on events at trot (-2ms for forelimbs and 1ms for hindlimbs) are small enough that the methods would be reliable for measuring such changes”*  Later in the discussion, when we evaluate the usefulness of our methods for calculating stance duration, we have similarly added details of lameness-related changes reported in the literature to give biological context:  “*In the literature, it was reported that unilateral forelimb lameness increased the stance phase of the ipsilateral hindlimb by 1.3% and that of both forelimbs by 2.3% in horses trotting on a treadmill* (12)*. The mean error in trotting stance durations calculated using the described methods range from 0.1 to 1.16% of a stride duration, suggesting that the methods are sufficiently accurate to detect these lameness-dependent changes.*” |
| **5** | The sample frequency may also be a limitation here if there is any intention to validate for faster gaits later on- 200Hz is likely limiting for detecting subtle variations at the gallop. | We agree that this is a valid point. Nonetheless, for this paper, gallop was not our focus and, in the literature, 200Hz has been widely accepted as a sufficient frequency for capturing the features of trot in both sound and lame horses (3) (5) (17). |
| **Reviewer 2- Specific Comments** | | |
| **1** | Line 55-58 – Consider re-wording, as the use of the pastern sensor and the fact the methods are not available aren’t quite clear | Thank you. We have updated the text to read:  *“Several previous studies have used inertial devices attached at the level of the pasterns to identify strides* (2) (3) (4)*, and there is a commercial system which uses these methods (Lameness Locator™, Equinosis, Columbia). However, to the authors’ best knowledge, there are no published methods for specific gait event detection which are publicly available.”*  We hope that this informs the reader that there are no other publications in the public domain describing methods to detect gait events from a pastern-mounted sensor, whilst acknowledging the fact that previous authors (particularly Dr K. Keegan) have used pastern-mounted sensors (as per Reviewer 1’s comment). |
| **2** | Line 73 – Need to specify that the figures in brackets (presumably) indicate standard deviation | The text has been updated to read:  *“Eleven horses (eight geldings and three mares, of mean(sd) heights and ages of 154(21)cm and 12(8)years)”* |
| **3** | Line 82 – Ideally use anatomical terms the first time the locations of sensors are specified (e.g., pasterns (proximal phalanges), cannon bones (third metacarpal bones)) as lay nomenclature may vary globally. | A good point- the text has been updated to read:  “*Sensors were attached laterally to the hooves and the regions of the pasterns (proximal phalanges) and cannon bones (third meta-carpal and -tarsal bones) (Fig 1) of the left fore- and hindlimbs*.” |
| **4** | Line 88 – fore- and hindlimbs | These alterations have been made, thank you |
| **5** | Line 91- Were the surfaces level? If the information is available, it would also be helpful to specify how firm the grass surface was as there can be considerable variation which would likely affect the accuracy of the sensors. It would also be helpful to specify if there was there any variation in the grass surface between trials, or if the trials were all performed on the same day. | Thank you for this comment. The trials for each individual horse were carried out during the same recording session but trials from all horses were not all collected on the same day. However, efforts were made to ensure similar conditions for all horses. All the test areas were level. Data was always collected on a dry day, when there had been little rainfall, meaning the grass surface was firm. The grassland used was grazing pasture and the grass was always short. The artificial surface (‘sand’) was well maintained and of uniform sand and rubber chip composition.  To inform readers of the paper, we have added the lines:  “*Efforts were made to ensure data was recorded under similar conditions for all horses. All the surfaces were level and datasets were collected during periods of dry weather when the grass field would be firm and the sand soft*.” |
| **6** | Line 101 – please double check manufacturer citation, I think this should be The MathWorks not MathWorks. | Thank you. The citation has been updated, As per PLOS ONE’s referencing criteria, to:  “*MATLAB (version 2020R, The MathWorks Inc., Natick, Massachusetts, USA)*” |
| **7** | Line 106- fore- and hindlimb (please check throughout) | These alterations have been made, thank you |
| **8** | Line 108 – Add a citation here to make it clear if you are still referring to reference 4 | This has been done, thank you |
| **9** | Line 155 & after – consider trough instead of valley? | Suggestion implemented, thank you |
| **10** | Line 202 – why was p>0.01 used rather than p<0.05? | A more conservative threshold P-value of 0.01, rather than 0.05, was chosen due to the small sample size. Choosing a lower p-value reduces the risk of incurring a Type I error. |
| **11** | Lines 230 – 232 – consider adding ‘respectively’ to sentence for clarity | We have updated the text to:  “*A total of 1465 walk and 1255 trot strides were analysed. These were 500, 535 and 430 walk strides and 438, 399 and 418 trot strides on asphalt, grass and sand, respectively*”  And hope that this is clearer for the reader. |
| **12** | Line 254 – Specify if figure in brackets is SD the first time you use it in this section | When we first use this structure in the section, we have now used the wording:  *“…the two methods were equally successful with M1_p_ detecting events slightly late by a mean(sd) delay of 2(10)ms, and M2_p_ slightly early, -1(10)ms…”*  Where we use the mean(sd) structure in an earlier section, we have now also added clarification:  “*Eleven horses (eight geldings and three mares, of mean(sd) heights and ages of 154(21)cm and 12(8)years)*” |
| **13** | Line 314 – I agree that some movement relative to the horse is inevitable. Have you or others quantified the amount of sensor movement when attached at the cannon/pastern? If so please cite/specify this. | Thank you for this very interesting point. Having looked, we were unable to find any relevant data in the literature and have not carried out such analyses, ourselves. However, this is something which we would expect to be heavily dependent on the attachment method used and, thus, difficult to replicate paper to paper. |
| **14** | Line 377-378 – The hoof mounted technique has been used elsewhere at a variety of gaits – check Witte et al. 2004 | Thank you for this observation. The specific data processing method we used as the reference (7) was only validated by the authors on a hard surface. However, we believe that the paper you suggest (1) further justifies our use of Tijssen’s methods for data collected on the soft surface, as Witte et al. were successfully able to manually identify the hoof-on and -off events from the accelerations. We have added this justification to the limitations section of the manuscript:  “*However, a previous study which used a hoof-mounted accelerometer to record accelerations* (1)*, found that gait events could be manually selected from signals recorded on both hard and soft surfaces. Furthermore, visual inspection of Acc_R_ and AngVel_R_ recorded from the hooves on the softer surfaces in the present study, revealed that they were comparable to those recorded on asphalt, with similar peaks prominent*.” |
| **15** | Line 400 – typo for ‘event’ | Thank you |

# References

| 1. | Witte TH, Knill K, Wilson AM. Determination of peak vertical ground reaction force from duty factor in the horse (Equus caballus). J of Exp Biol. 2004; 207: 3639-48. |
| --- | --- |
| 2. | McCracken MJ, Kramer J, Keegan KG, Lopes M, Wilson DA, Reed SK, et al. Comparison of an inertial sensor system of lameness quantification with subjective lameness evaluation. Equine Vet J. 2012; 44: 652-6. |
| 3. | Keegan KG, Wilson DA, Kramer J, Reed SK, Yonezawa Y, Maki H, et al. Comparison of a body-mounted inertial sensor system–based method with subjective evaluation for detection of lameness in horses. American J of Vet Research. 2013; 74(1): 17-24. |
| 4. | Rhodin M, Roepstorff L, French A, Keegan KG, Pfau T, Egenvall A. Head and pelvic movement asymmetry during lungeing in horses with symmetrical movement on the straight. Equine Vet J. 2016; 48: 315-20. |
| 5. | Bragança FM, Bosch S, Voskamp JP, Marin-Perianu M, Van der Zwaag BJ, Vernooij JCM, et al. Validation of distal limb mounted inertial measurement unit sensors for stride detection in Warmblood horses at walk and trot. Equine Vet J. 2017: p. 545-51. |
| 6. | Sapone M, Martin P, Mansour KB, Château H, Marin F. Comparison of trotting stance detection methods from an inertial measurement unit mounted on the horse's limb. Sensors. 2020; 20(10): 2983. |
| 7. | Tijssen M, Hernlund E, Rhodin M, Bosch S, Voskamp JP, Nielen M, et al. Automatic hoof-on and -off detection in horses using hoof-mounted inertial measurement unit sensors. PLoS ONE. 2020 June; 15(6): e0233266. |
| 8. | Serra Bragança FM, Hernlund E, Thomsen MH, Waldern NM, Rhodin M, Byström A, et al. Adaptation strategies of horses with induced forelimb lameness walking on a treadmill. Equine Vet J. 2020; 00: 1-12. |
| 9. | Clayton H. Classification of collected trot, passage and piaffe based on temporal variables. Equine Vet J Suppl. 1997; 23: 54-7. |
| 10. | Holström M, Fredricson I, Drevemo S. Biokinematic differences between riding horses judged as good and poor at the trot. Equine Vet J Suppl. 1994; 17: 51-6. |
| 11. | Clayton H. Cinematographic analysis of the gait of lame horses. J Equine Vet Sci. 1986; 6: 70-8. |
| 12. | Buchner HHF, Savelberg HHCM, Schamhardt HC, Barneveld A. Temporal stride patterns in horses with experimentally indiced fore- or hindlimb lameness. Equine Vet J. 1995; 18: 161-5. |
| 13. | Weishaupt MA, Wiestner T, Hogg HP, Jordan P, Auer JA. Compensatory load redistribution of horses with induced weight-bearing forelimb lameness trotting on a treadmill. Vet J. 2006; 171(1): 135-46. |
| 14. | Bragança FM, Hernlund E, Thomsen MH, Waldern NM, Rhodin M, Byström A, et al. Adaptation strategies of horses with induced forelimb lameness walking on a treadmill. Equine Veterinary Journal. 2020;: 1-12. |
| 15. | Self Davies ZT, Spence AJ, Wilson AM. External mechanical work in the galloping racehorse. Biol Lett. 2019; 15: 20180709. |
| 16. | Self Davies ZT, Spence AJ, Wilson AM. Ground reaction forces of overground galloping in ridden Thoroughbred racehorses. J of Exp Biol. 2019; 222(16): 204107. |
| 17. | Bosch S, Serra Bragança F, Marin-Perianu M, Marin-Perianu R, van der Zwaag BJ, Voskamp J, et al. EquiMoves: A wireless networked inertial measurement system for objective examination of horse gait. Sensors. 2018; 18(850). |
